# Gut microbiota functional remodeling and butyrate depletion amplify anti-Ro/La antibody-driven type I interferon activation in neonatal lupus

**DOI:** 10.1080/19490976.2026.2728464

**Published:** 2026-09-16

**Authors:** Wenqiang Sun, Yihui Li, Xue Liu, Shuyang Yu, Wenmei Li, Huawei Wang, Haifeng Geng, Lili Li, Jinhui Hu, Jie Huo, Wanyan Zhang, Jing Fu, Xinyun Jin, Heng Li, Xihui Zhou, Xueping Zhu

**Affiliations:** a Department of Neonatology, Children’s Hospital of Soochow University, Suzhou, China; b Suzhou Medical College, Soochow University, Suzhou, China; c Department of Microbiology, School of Basic Medical Sciences, Suzhou Medical College, Soochow University, Suzhou, China; d Department of Neonatology, the Affiliated Suzhou Hospital of Nanjing Medical University, Suzhou, China; e Neonatal Medical Center, Huai'an Maternal and Child Health Care Center, Huai'an, China; f Department of Neonatology, Yangzhou Maternity and Child Health Care Hospital, Yangzhou, China; g Department of Pediatrics, The First Affiliated Hospital of Xi'an Jiaotong University, Xi'an, China; h Department of Obstetrics and Gynecology, The First Affiliated Hospital of Xi'an Jiaotong University, Xi'an, China; i Department of Pediatric Infectious Diseases, Children’s Hospital of Soochow University, Suzhou, China

**Keywords:** Butyrate, gut microbial metabolism, neonatal lupus erythematosus, shotgun metagenomics, type I interferon

## Abstract

The early-life gut microbiome may influence susceptibility to antibody-mediated neonatal autoimmunity, but the underlying mechanisms remain poorly understood. We investigated whether gut microbial functional capacity and metabolites influence autoantibody-dependent immune activation in 90 neonates, including healthy controls, anti-Ro/La-exposed neonates without neonatal lupus erythematosus (No-NLE), and neonates with NLE (*n* = 30 per group). Shotgun metagenomic profiling demonstrated progressive remodeling of the neonatal gut microbiome across the three groups, with anti-Ro/La exposure associated with depletion of early-life commensal-associated taxa, including *Bifidobacterium*, *Rothia*, and *Clostridium*, and enrichment of taxa with opportunistic potential, including *Klebsiella* and *Enterococcus*, with greatest ecological divergence in neonates with NLE. Functional profiling identified altered microbial carbohydrate-processing capacity, marked by enrichment of glycosyltransferase family 4 (GT4) and depletion of GT2 in NLE. These alterations coincided with broad reductions in plasma short-chain fatty acid metabolites, most prominently butyrate, together with increased serum immunoglobulin G (IgG) and interferon-*α* (IFN-*α*) and decreased complement component 4 (C4). A GT4–*Klebsiella*–*Rothia*–IFN-*α* signature distinguished NLE from No-NLE (AUC = 0.883; 95% CI, 0.799–0.967). In functional assays, pooled bacteria-depleted fecal filtrates from neonates with NLE potentiated IFN-*α* production by neonatal peripheral blood mononuclear cells in the presence of anti-Ro/La-positive plasma. Conversely, sodium butyrate suppressed anti-Ro/La-associated IFN-*α* production and reduced 28 inflammation-related proteins, including CXCL10, ADA, and PD-L1, involved in cytokine, IL-17, and TNF signaling. Together, these findings provide functional evidence supporting a microbiota-associated butyrate–type I interferon pathway that may amplify maternal autoantibody-dependent immune activation and contribute to the clinical manifestation of NLE.

## 1. Introduction

Neonatal lupus erythematosus (NLE) is a passively acquired autoimmune disorder resulting from the transplacental transfer of maternal autoantibodies, predominantly antibodies against Ro/Sjögren syndrome-related antigen A (SSA) and/or La/Sjögren syndrome-related antigen B (SSB).^1-4^ Its clinical manifestations are heterogeneous and may involve the skin, hematologic and hepatobiliary systems, and cardiac conduction system, with congenital atrioventricular block representing the most severe and irreversible complication.^5-7^ Although maternal autoantibody exposure is necessary for disease development, only a subset of exposed neonates develops clinical manifestations.^2^ This incomplete penetrance suggests that additional neonatal or environmental factors influence whether maternal autoantibody exposure progresses to clinically apparent disease.

The early postnatal period represents a critical window in which gut microbial colonization and immune maturation occur concurrently. During this period, delivery mode, feeding practices, maternal immune status, and other perinatal exposures substantially shape the developing gut microbiome.^8-12^ In turn, the gut microbiome contributes to immune development by regulating epithelial barrier function, inflammatory responses, and immune tolerance, while alterations in the host immune environment can reciprocally reshape microbial community structure and function. Microbiota-derived metabolites provide an important interface in this bidirectional interaction. In particular, short-chain fatty acids (SCFAs) can regulate immune-cell metabolism, epigenetic programs, cytokine responses, and inflammatory signaling.^13-18^ Early-life variation in microbial community function and metabolite availability may therefore modify the threshold and magnitude of neonatal immune responses to maternally transferred autoantibodies.

Advances in shotgun metagenomics and functional annotation have shifted microbiome research beyond compositional profiling toward the characterization of microbial genes, metabolic pathways, and bioactive potential.^8^ ^,^ ^19^ ^,^ ^20^ However, despite growing evidence for interactions among the maternal immune environment, the developing microbiome, and neonatal immunity, the microbial functions that modify immune responses following maternal autoantibody exposure remain poorly understood.^16^ ^,^ ^21^ In particular, it remains unclear whether functional remodeling of the early-life gut microbiome and altered microbiota-associated metabolite availability modulate autoantibody-dependent type I interferon activation and thereby contribute to the differential clinical expression observed among anti-Ro/La-exposed neonates.

To address these questions, we integrated shotgun metagenomics, plasma metabolomics, immunological profiling, and in vitro functional assays in healthy neonates, anti-Ro/La-exposed neonates without NLE, and neonates with NLE. We first characterized taxonomic and functional remodeling of the gut microbiome and examined its relationships with circulating metabolites and immune phenotypes. We then tested whether bacteria-depleted fecal filtrates from the three neonatal groups differentially modulated IFN-*α* production by neonatal peripheral blood mononuclear cells in the presence of anti-Ro/La-positive plasma. Finally, guided by the observed metabolic alterations, we evaluated whether butyrate could suppress anti-Ro/La-associated IFN-*α* activation and the accompanying inflammatory protein response. Through this integrated approach, we sought to functionally interrogate a microbiota–metabolite–type I interferon axis that may modify autoantibody-dependent immune activation and contribute to the heterogeneous clinical expression of NLE.

## 2. Methods

### 2.1. Study population and diagnostic criteria

We conducted a multicenter, observational case–control study between January 1, 2024, and May 31, 2025, at five tertiary medical centers in China: the Children’s Hospital of Soochow University, the First Affiliated Hospital of Xi’an Jiaotong University, the Affiliated Suzhou Hospital of Nanjing Medical University, Huai’an Maternal and Child Health Care Center, and Yangzhou Maternity and Child Health Care Hospital. The study protocol was approved by the Ethics Committee of the Children’s Hospital of Soochow University (approval no. 2023CS024) and by the institutional ethics committees of the other participating centers. The study was conducted in accordance with the Declaration of Helsinki of 1975, as revised in 2008. Written informed consent was obtained from the legal guardians of all participating neonates before enrollment.

Pregnancies at potential risk of NLE were identified antenatally or during the perinatal period on the basis of maternal autoimmune disease and/or positivity for anti-SSA/Ro or anti-SSB/La antibodies. Eligible neonates were enrolled at birth and underwent standardized clinical evaluation. NLE was diagnosed when both of the following criteria were met ^2^ ^,^ ^3^: (1) detection of at least one anti-SSA/Ro or anti-SSB/La antibody in maternal or neonatal serum; and (2) the presence of clinical manifestations compatible with NLE. For consistency, exposure to anti-SSA/Ro and/or anti-SSB/La antibodies is collectively termed “anti-Ro positivity” throughout this manuscript. Clinical classification was based on established diagnostic criteria for neonatal lupus and incorporated cutaneous, hematologic, gastrointestinal, and cardiac manifestations, together with relevant laboratory and imaging findings. Anti-SSA/Ro and anti-SSB/La antibodies were measured using chemiluminescence immunoassays, and the reported anti-SSA/Ro and anti-SSB/La reactivity levels were classified semi-quantitatively as negative, +, ++, or +++.

### 2.2. Study grouping and follow-up

Neonates fulfilling the diagnostic criteria for NLE were assigned to the NLE group. For each NLE case, one anti-SSA/Ro- and/or anti-SSB/La-positive neonate born during the same recruitment period and without NLE-related manifestations was selected for the No-NLE group. Matching was performed at a 1:1 ratio according to gestational age and birth weight. Healthy controls were neonates born to mothers without a known autoimmune disease or documented anti-Ro/La positivity and without clinical features suggestive of NLE.

All enrolled neonates were followed until 6 months of age to comprehensively assess the occurrence of NLE-related clinical manifestations and to exclude potential late-onset NLE in the No-NLE group. The presence of NLE-related clinical features was independently evaluated by two clinicians who were unaware of microbiome, metabolomic, and immunological results. In cases of disagreement, adjudication was performed by a third senior specialist. Neonates for whom a consensus diagnosis could not be reached after multidisciplinary review were excluded to minimize misclassification bias. Final classification into the control, No-NLE, and NLE groups was determined according to maternal or neonatal antibody exposure and the presence or absence of NLE-related manifestations during follow-up. Group labels were finalized before metagenomic, metabolomic, immunological, and functional analyzes were performed. The temporal sequence of participant enrollment, biospecimen collection, longitudinal clinical follow-up, final group adjudication, and downstream analyzes is summarized in Supplementary **Figure S1**.

### 2.3. Clinical data and biological sample collection

Maternal and neonatal clinical information was recorded using standardized case report forms. Neonatal variables included sex, gestational age, birth weight, mode of delivery, premature rupture of membranes, postnatal asphyxia, and feeding practices. Maternal variables included age, autoimmune disease diagnosis, medication exposure during pregnancy, and anti-SSA/Ro and anti-SSB/La antibody status.

All neonatal blood and fecal samples were collected between postnatal days 7 and 10 according to a prespecified protocol. Sampling was completed before the end of longitudinal follow-up and before final clinical adjudication. None of the neonates had received antibiotics or probiotic supplementation before sample collection. Peripheral venous blood was collected and centrifuged for serum separation. Fecal samples were obtained using sterile collection procedures. All specimens were processed and transferred to −80°C storage within 2 hours of collection. Neonatal anti-SSA/Ro and anti-SSB/La antibodies were measured in serum obtained during the early postnatal sampling period.

### 2.4. Fecal microbial deoxyribonucleic acid (DNA) extraction, metagenomic sequencing, and analysis

Genomic DNA was extracted from neonatal fecal samples and subjected to shotgun metagenomic sequencing using the DNA nanoball sequencing (DNBSEQ) high-throughput sequencing platform with paired-end 150-base-pair reads (PE150). Sequencing reads were quality-filtered, and host-derived sequences were removed before downstream analysis. High-quality reads were assembled to construct a nonredundant microbial gene catalog, followed by taxonomic and functional annotation. Microbial taxonomic profiles were generated using a lowest common ancestor (LCA)-based approach against the National Center for Biotechnology Information (NCBI) nonredundant (NR) protein database. Community diversity, differential microbial taxa, and microbial co-occurrence networks were analyzed to characterize microbiome alterations associated with NLE. Functional profiles were annotated using the Kyoto Encyclopedia of Genes and Genomes (KEGG) and Carbohydrate-Active enZYmes (CAZy) databases, with particular emphasis on carbohydrate-active enzyme (CAZyme) families. Differential microbial taxa and functional features were identified using linear discriminant analysis effect size (LEfSe) analysis. Detailed procedures for DNA extraction, library preparation, sequencing quality control, gene catalog construction, taxonomic annotation, functional annotation, and analytical parameters are provided in **Appendix A**.

### 2.5. Plasma metabolomics analysis

Plasma metabolomics analysis was performed on plasma samples randomly selected from the primary metagenomic cohort (*n* = 8 per group). Untargeted metabolomic profiling was conducted using ultra-high-performance liquid chromatography coupled with high-resolution mass spectrometry (UHPLC-HRMS) on an Orbitrap Exploris^TM^ 700 platform in both positive and negative electrospray ionization modes. Raw mass spectrometry data were processed for peak detection, alignment, metabolite annotation, and normalization to minimize systematic variation before statistical analysis. Global metabolic differences between groups were assessed using orthogonal partial least squares discriminant analysis (OPLS-DA). The robustness of the OPLS-DA models was evaluated by permutation testing. Differential metabolites were identified based on variable importance in projection (VIP) scores and fold change thresholds (VIP > 1 and fold change ≥ 2 or ≤ 0.5). Hierarchical clustering analysis was subsequently performed to visualize metabolite expression patterns across samples. Differential metabolites were further subjected to KEGG pathway annotation and enrichment analysis to identify significantly altered metabolic pathways.

### 2.6. Measurement of humoral and complement immune parameters and interferon-*α* (IFN-*α*)

Serum concentrations of immunoglobulin G (IgG), immunoglobulin A (IgA), immunoglobulin M (IgM), and complement components 3 (C3) and 4 (C4) were quantitatively measured using a fully automated specific protein analysis system with commercially available reagents, following the manufacturers’ instructions. All measurements were performed on the Beckman IMMAGE 8000 specific protein analyzer (Beckman Colter, United States). Serum IFN-*α* levels were determined using a high-sensitivity human IFN-*α* enzyme-linked immunosorbent assay (ELISA) kit (BMS216, Thermo Fisher Scientific). Quantification was performed using standard curves, with quality-control samples included in each assay. All samples were measured in duplicate, and laboratory personnel were blinded to study group assignments throughout the experimental procedures.

### 2.7. Preparation of fecal filtrates and in vitro peripheral blood mononuclear cell (PBMC) stimulation assays

Frozen fecal samples were thawed under sterile conditions. For each PBMC donor, a new and independent fecal pool was prepared for each study group by randomly combining equal wet-weight amounts of three fecal samples, with no sample reused across pools. Each pooled sample was suspended in sterile phosphate-buffered saline (PBS) at 10% (weight/volume [w/v]), homogenized, centrifuged at 12,000 × g for 5 min, and filtered through a 0.22 μm membrane to obtain a bacteria-depleted fecal filtrate. PBMCs were isolated from umbilical cord blood of six independent healthy neonatal donors and seeded at a density of 2.5 × 10^5^ cells per well in 96-well flat-bottom culture plates. PBMCs from each donor were tested under all experimental conditions. Cells were co-incubated with fecal filtrates at 0.1% (volume/volume [v/v]), corresponding to a final fecal-equivalent concentration of 0.01% (w/v), and plasma at 0.1% (v/v). Anti-Ro/La-positive plasma and healthy neonatal plasma were each prepared by pooling equal volumes from three donors. To enhance IFN-*α* induction, freeze–thaw–generated necrotic cell supernatants were added to the culture system, as previously described.^22^ Cells were incubated at 37°C in a humidified atmosphere containing 5% CO₂ for 24 h, after which culture supernatants were collected for subsequent analyzes. The experimental design compared the effects of Ctrl-, No-NLE-, and NLE-derived fecal filtrates on neonatal PBMC responses under healthy or anti-Ro/La-positive plasma backgrounds, with PBS serving as the fecal filtrate blank.

### 2.8. Butyrate intervention experiments

To evaluate the effect of butyrate on anti-Ro/La-positive plasma-associated IFN-*α* activation, PBMCs were isolated from the umbilical cord blood of eight independent healthy neonatal donors. PBMCs from each donor were tested under both vehicle-control and sodium-butyrate conditions. PBMCs were pretreated for 30 min with sodium butyrate (Sigma-Aldrich, Cat. No. B5887; final concentration, 1 mM) or an equal volume of sterile PBS as the vehicle control. Cells were subsequently stimulated with the pooled anti-Ro/La-positive neonatal plasma (0.1% [v/v]) and freeze–thaw-generated necrotic cell supernatant. Sodium butyrate was not removed after pretreatment and remained present throughout the subsequent 24-h stimulation period. Cells were incubated at 37°C in a humidified atmosphere containing 5% CO₂ for 24 h, after which culture supernatants were collected for IFN-*α* measurement and Olink targeted proteomic profiling. The 1 mM sodium butyrate concentration was selected based on preliminary dose-ranging experiments.

### 2.9. Olink proteomic analysis

Targeted proteomic profiling was performed on PBMC culture supernatants obtained from the butyrate intervention experiments using the Olink Target 96 Inflammation panel based on proximity extension assay technology. Protein abundance was reported as Normalized Protein eXpression (NPX) values on a log_2_ scale following preprocessing and quality control using Olink NPX Manager. Differences in protein abundance between the butyrate-treated and vehicle-control groups were evaluated using two-sided paired t-tests. Differentially expressed proteins were subsequently subjected to KEGG pathway enrichment analysis to characterize biological pathways associated with butyrate-mediated modulation of anti-Ro/La-induced inflammatory responses.

### 2.10. Statistical Analysis

All statistical analyzes were performed using R software (version 4.2.1), and figures were generated using the ggplot2 package. Continuous variables are presented as mean ± standard deviation or median with interquartile range, as appropriate, and categorical variables as counts and percentages. Data distribution was assessed before statistical testing. Normally distributed variables were analyzed using parametric tests, whereas non-normally distributed variables were analyzed using non-parametric tests. Two-group comparisons were performed using two-sided t-tests or Wilcoxon rank-sum tests, and categorical variables were compared using chi-square or Fisher’s exact tests. Comparisons among three groups were performed using Kruskal–Wallis tests followed by two-sided pairwise Wilcoxon rank-sum tests.

Maternal anti-SSA/Ro and anti-SSB/La antibody reactivity was analyzed as an ordinal variable. Reactivity grades reported by the clinical laboratory system (+, ++, +++) were encoded as 1, 2, and 3, respectively, whereas antibody-negative samples were assigned a value of 0. A combined maternal anti-Ro/La burden score was calculated by summing anti-SSA/Ro and anti-SSB/La reactivity grades. Ordered differences in antibody reactivity categories across groups were assessed using the Cochran–Armitage trend test. Correlations among maternal antibody burden, microbial features, metagenomic functional profiles, metabolites, and immune parameters were evaluated using Spearman’s rank correlation analysis.

Receiver operating characteristic (ROC) curve analyzes were performed to evaluate the discriminatory performance of microbial, functional, and immune markers between NLE and No-NLE groups. Candidate predictors were selected using least absolute shrinkage and selection operator (LASSO) regression with cross-validation and the lambda.1se criterion. Selected variables were subsequently entered into multivariable logistic regression models adjusted for relevant clinical covariates, including gestational age, delivery mode, and feeding practices. Model discrimination was assessed using area under the curve (AUC) values with 95% confidence intervals, and a nomogram was constructed based on independent predictors. All statistical tests were two-sided, and *P* < 0.05 was considered statistically significant.

## 3. Results

### 3.1. Clinical characteristics of the study population

A total of 90 neonates were enrolled in this study, including 30 healthy neonates and 60 neonates born to mothers positive for anti-Ro antibodies. The latter were further stratified into a No-NLE group and an NLE group, with 30 neonates in each. The clinical characteristics of the study population are summarized in **Table S1**. No significant differences were observed among the three groups in baseline clinical characteristics (*P* > 0.05). Among anti-Ro-positive neonates, the No-NLE and NLE groups showed comparable maternal disease profiles and prenatal immunomodulatory medication exposure (*P* > 0.05). Compared with the No-NLE group, the NLE group showed a significant shift toward higher maternal and neonatal anti-SSA/Ro and anti-SSB/La reactivity grades, as demonstrated by Cochran–Armitage trend analysis (all *P* < 0.05) (**Table S2**).

### 3.2. Anti-Ro exposure-associated remodeling of the neonatal gut microbiome

**Figure 1.**
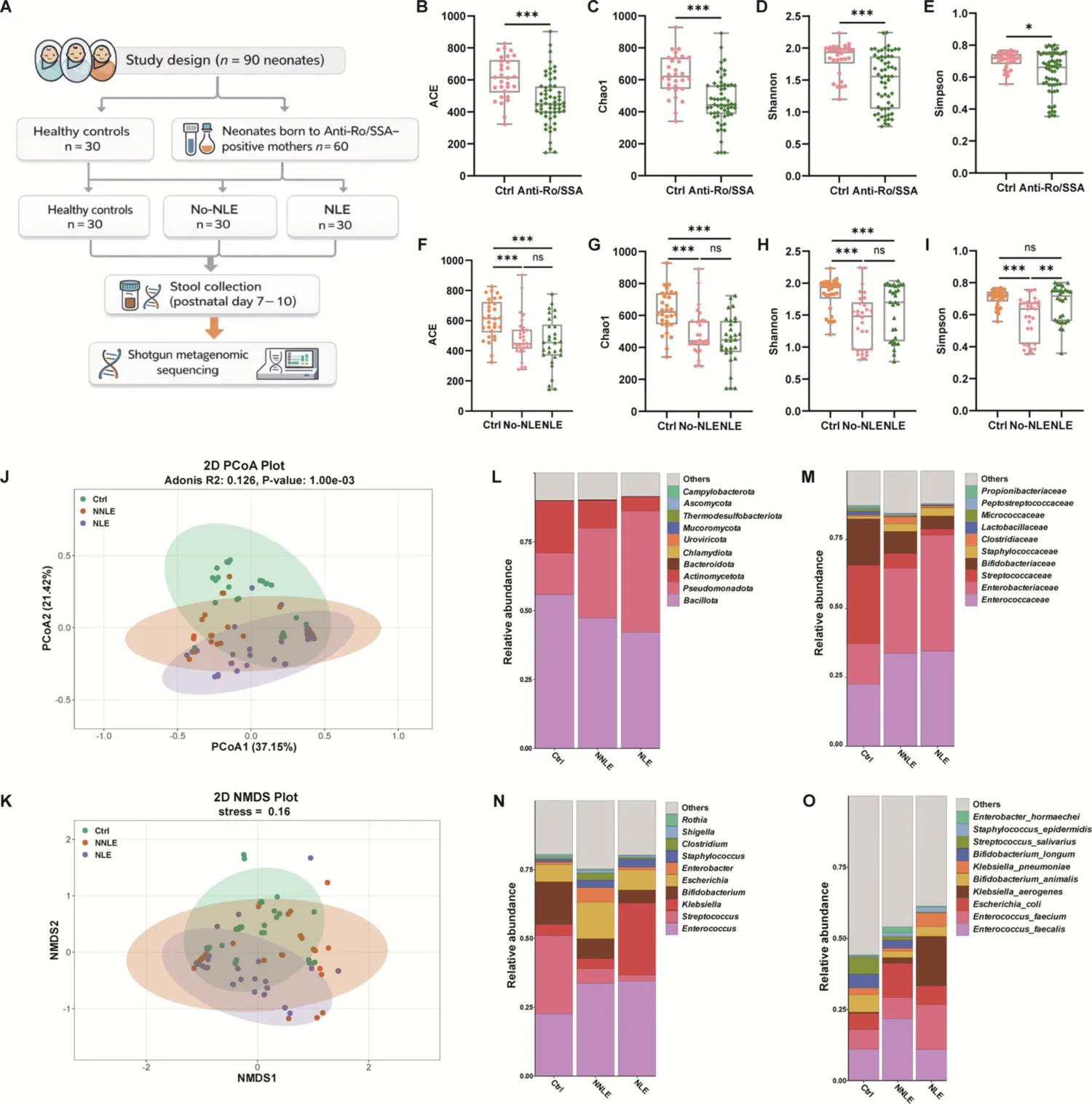
Alpha diversity, beta diversity, and taxonomic composition of the neonatal gut microbiome. (A) Overview of the study groups and workflow. (B-I) Alpha-diversity indices (ACE, Shannon, Simpson, and Chao1) in healthy controls (Ctrl; *n* = 30), anti-Ro/La antibody-exposed neonates without NLE (No-NLE; *n* = 30), and neonates with NLE (*n* = 30). Two-group comparisons used the Wilcoxon rank-sum test, whereas three-group comparisons used the Kruskal-Wallis test followed by pairwise Wilcoxon rank-sum tests. (J, K) Principal coordinates analysis (PCoA) and non-metric multidimensional scaling (NMDS) based on Bray-Curtis dissimilarities. Group differences in community structure were assessed by permutational multivariate analysis of variance (PERMANOVA). (L-O) Relative-abundance profiles of the gut microbiome at the phylum, family, genus, and species levels, respectively. Only the most abundant taxa are displayed; remaining taxa are grouped as “Others.” All statistical tests were two-sided. *P < 0.05, **P < 0.01, ***P < 0.001. ACE, abundance-based coverage estimator; NLE, neonatal lupus erythematosus.

To characterize microbiome alterations associated with maternal anti-Ro/La antibody exposure and NLE manifestation, shotgun metagenomic sequencing was performed on fecal samples from healthy controls and anti-Ro/La-exposed neonates (Figure 1A). Alpha diversity analyzes demonstrated reduced microbial richness and diversity in anti-Ro/La-exposed neonates, with significantly lower ACE, Chao1, Shannon, and Simpson indices compared with healthy controls (Figure 1B–E). Further three-group comparisons revealed that alpha diversity was significantly lower in both the No-NLE and NLE groups than in the Ctrl group. Notably, the Simpson index was significantly higher in the NLE group than in the No-NLE group, whereas no significant differences were observed between these two groups for the Shannon, ACE, or Chao1 indices (Figure 1F–I). Beta diversity analyzes based on principal coordinates analysis (PCoA) and non-metric multidimensional scaling (NMDS) demonstrated distinct microbial community structures among the three groups (Figure 1J, K). Taxonomic profiling revealed a progressive shift in microbial composition from healthy controls to No-NLE and subsequently NLE neonates (Figure 1L–O). At the phylum level, anti-Ro/La-exposed neonates showed decreased *Bacillota* and *Actinomycetota* and increased *Pseudomonadota* compared with controls. At the genus level, *Enterococcus* and *Staphylococcus* were enriched, whereas *Streptococcus* and *Bifidobacterium* were depleted. Comparison between anti-Ro/La-exposed neonates with and without NLE further identified disease-associated microbial signatures. NLE neonates exhibited enrichment of *Klebsiella*, whereas *Enterobacter* was preferentially increased in No-NLE neonates. At the species level, *Enterococcus faecium* and *Klebsiella aerogenes* were enriched, whereas *Bifidobacterium longum* was reduced in NLE neonates.

### 3.3. Identification of anti-Ro– and NLE-associated differential genera by high-resolution metagenomic analysis

**Figure 2.**
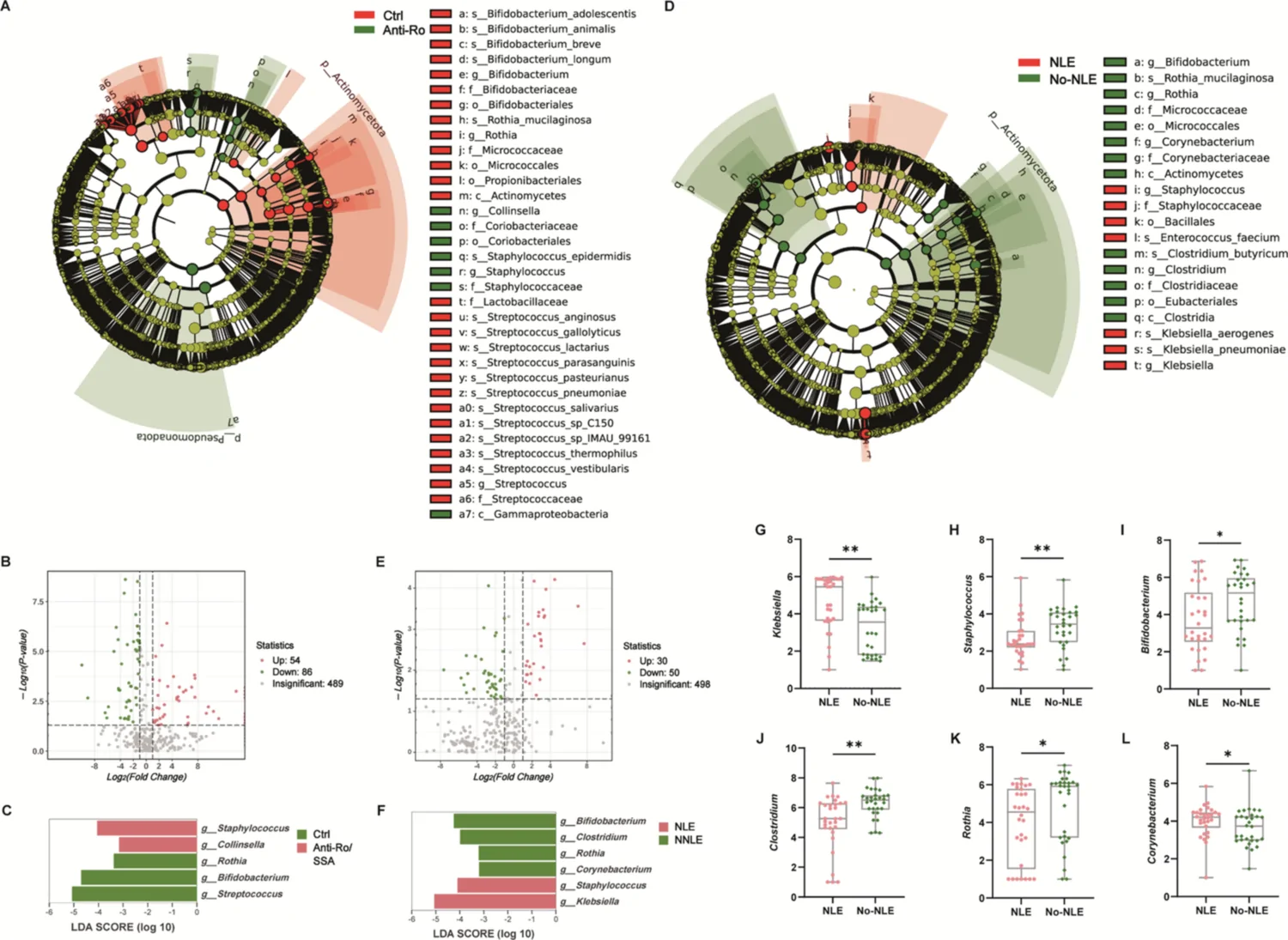
Differential gut microbial genera associated with anti-Ro/La antibody and NLE. (A–C) Differential taxonomic features between anti-Ro/La antibody-exposed neonates (*n* = 60) and healthy controls (Ctrl, *n* = 30), displayed as a LEfSe cladogram, volcano plot, and LDA score plot, respectively. (D–F) Differential taxonomic features between neonates with NLE (*n* = 30) and No-NLE neonates (*n* = 30), displayed as a LEfSe cladogram, volcano plot, and LDA score plot, respectively. LEfSe features were selected using an LDA score > 3.0 and *P* < 0.05. (G–L) Relative abundances of representative genera differing between the NLE and No-NLE groups. Values were log10-transformed and shifted to positive values for visualization. Two-group comparisons used the Wilcoxon rank-sum test. NLE, neonatal lupus erythematosus; LEfSe, linear discriminant analysis effect size; LDA, linear discriminant analysis; No-NLE, anti-Ro/La antibody-exposed neonates without NLE; *P < 0.05, **P < 0.01, ***P < 0.001.

To further define microbial taxa associated with anti-Ro/La exposure and NLE manifestation, genus-level differential abundance analyzes were performed using shotgun metagenomic profiles, revealing distinct microbial signatures across the Ctrl, No-NLE, and NLE groups **(Figure S2)**. Compared with healthy controls, anti-Ro/La-exposed neonates exhibited extensive microbial alterations, with 629 genera detected, including 54 enriched and 86 depleted genera (Figure 2A, B). Among these differential taxa, *Staphylococcus* and *Collinsella* were increased, whereas *Streptococcus*, *Bifidobacterium*, and *Rothia* were reduced in anti-Ro/La-exposed neonates (Figure 2C). To identify microbial features specifically associated with NLE development beyond antibody exposure alone, comparisons were performed between No-NLE and NLE groups. Among 578 detected genera, 30 were enriched and 50 were depleted in NLE neonates compared with No-NLE neonates (Figure 2D, E). LEfSe analysis identified *Klebsiella* and *Staphylococcus* as enriched taxa in the NLE group and *Bifidobacterium*, *Clostridium*, *Rothia*, and *Corynebacterium* as depleted taxa; and these differences were further supported by genus-level pairwise comparisons (Figure 2F–L).

### 3.4. Gut microbiome interaction networks and metabolic signatures

**Figure 3.**
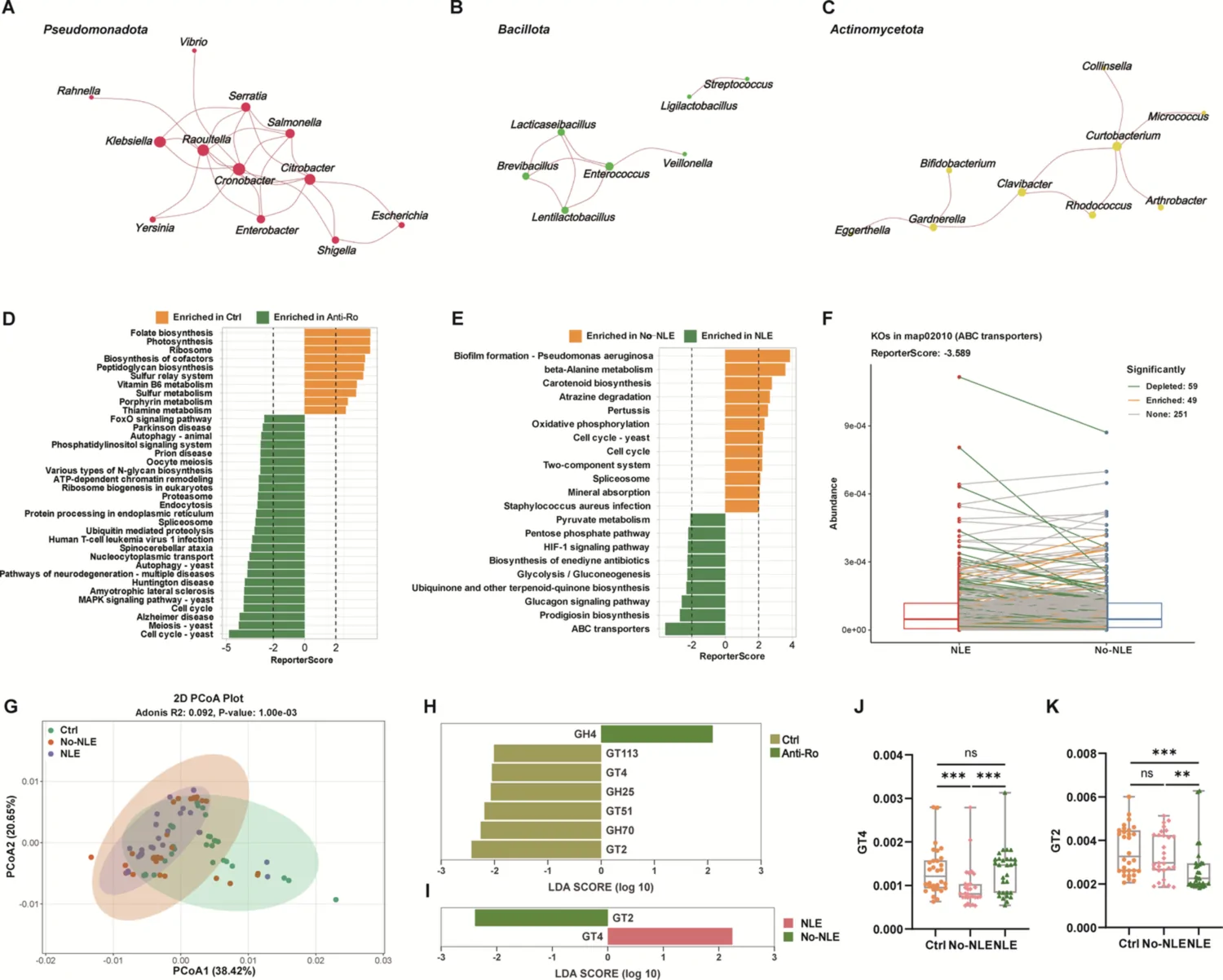
Altered microbial ecological networks and metagenomic functional profiles associated with anti-Ro/La antibody exposure and NLE. (A–C) Genus-level co-occurrence network analysis of differential microbial genera. Nodes represent genera and are sized according to relative abundance; edges represent microbial correlations. (D, E) KEGG pathway enrichment of differentially abundant genes between healthy controls and anti-Ro/La antibody-exposed neonates, and between NLE and No-NLE neonates, respectively. (F) Abundance of genes assigned to the ABC transporter pathway in NLE and No-NLE neonates. (G) PCoA of CAZyme profiles based on Bray-Curtis dissimilarities; group differences were assessed by PERMANOVA. (H, I) LEfSe analyzes of differential CAZyme families between healthy controls and anti-Ro/La antibody-exposed neonates, and between NLE and No-NLE neonates, respectively. LEfSe features were selected using an LDA score > 2.0 and *P* < 0.05. (J–K) Relative abundances of glycosyltransferase families GT4 and GT2 in the indicated groups. Three-group comparisons were performed using the Kruskal–Wallis test followed by pairwise Wilcoxon rank-sum tests. NLE, neonatal lupus erythematosus; KEGG, Kyoto Encyclopedia of Genes and Genomes; No-NLE, anti-Ro/La antibody-exposed neonates without NLE; ABC, ATP-binding cassette; PCoA, principal coordinates analysis; CAZyme, carbohydrate-active enzyme; PERMANOVA, permutational multivariate analysis of variance; GT, glycosyltransferase. *P < 0.05, **P < 0.01, ***P < 0.001.

To investigate whether microbial alterations were accompanied by functional changes, microbial interaction networks and metagenomic functional profiles were analyzed (Figure 3). The results showed that these differential genera were predominantly distributed within *Pseudomonadota*, *Bacillota*, and *Actinomycetota* and exhibited markedly reshaped co-occurrence relationships (Figure 3A–C). KEGG pathway analysis showed broad functional differences between anti-Ro-positive neonates and controls, involving metabolism, immune-related pathways, and cellular processes (Figure 3D). Compared with No-NLE neonates, NLE neonates showed enrichment of ATP-binding cassette (ABC) transporter-related pathways and carbohydrate metabolism-associated functions (Figure 3E, F). Consistent with these findings, CAZy profiling revealed distinct carbohydrate metabolic potential among the three groups (Figure 3G). LEfSe analysis revealed that, compared with the control group, the anti-Ro group exhibited a significant increase in glycoside hydrolase family 4 (GH4) abundance, along with marked reductions in multiple glycosyltransferase (GT) and GH families. Within the anti-Ro group, the NLE group was characterized by increased GT4 and decreased GT2 abundance (Figure 3H–K).

### 3.5. Immune dysregulation and microbiome–antibody associations in NLE

**Figure 4.**
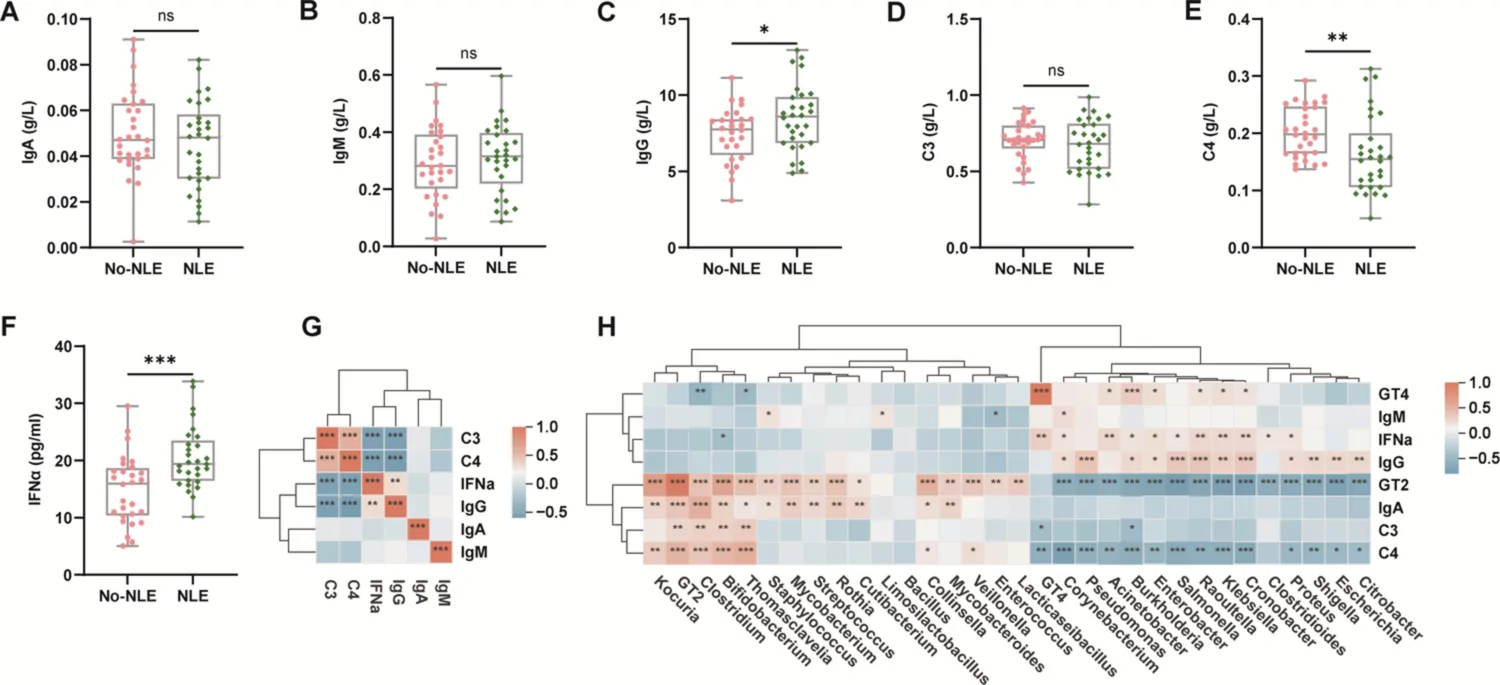
Humoral immune and complement profiles and their associations with gut microbial genera in NLE. (A–F) Serum concentrations of immunoglobulin A (IgA), IgM, IgG, complement components 3 (C3) and 4 (C4), and IFN-*α* in No-NLE (*n* = 30) and NLE (*n* = 30) neonates. Two-group comparisons were performed using two-sided t-tests or Wilcoxon rank-sum tests. (G) Spearman correlation matrix among serum immune and complement variables. (H) Spearman correlations between differentially abundant gut microbial genera and immune-related variables. NLE, neonatal lupus erythematosus; IFN-*α*, interferon-α; No-NLE, anti-Ro/La antibody-exposed neonates without NLE; *P < 0.05, **P < 0.01, ***P < 0.001.

Previous studies have indicated that humoral and complement immune responses contribute to NLE pathogenesis, with IFN-*α* representing a key inflammatory mediator. To determine whether NLE-associated microbial alterations were accompanied by immune dysregulation, humoral and complement-related parameters were compared between NLE and No-NLE neonates (Figure 4A–F). No significant differences were observed in IgA, IgM, or complement C3 levels (*P* > 0.05), whereas the NLE group exhibited significantly increased IgG levels and reduced complement C4 levels (*P* < 0.05). Serum IFN-*α* levels were also significantly elevated in NLE neonates compared with No-NLE neonates (*P* < 0.05). Correlation analyzes revealed varying degrees of association among IgG, IgA, IgM, C3, C4, and IFN-*α* levels (Figure 4G). Further examination of the relationships between gut microbial genera and these immune and complement parameters showed that multiple genera were significantly correlated with IgG, IgA, IgM, C3, C4, and IFN-*α* levels, displaying a clear taxonomic clustering of potentially beneficial versus potentially harmful taxa (Figure 4H). Specifically, genera enriched in NLE neonates, including inflammation-associated taxa, showed positive correlations with elevated IFN-*α* and IgG levels, whereas several taxa associated with healthier microbial profiles exhibited inverse correlations with these immune parameters. Maternal anti-Ro/La antibody burden was positively associated with *Klebsiella*, GT4 abundance and IFN-*α*, whereas *Bifidobacterium* and GT2 showed inverse associations (**Table S3**).

### 3.6. Integrated microbial and immunological signatures predict NLE risk

**Figure 5.**
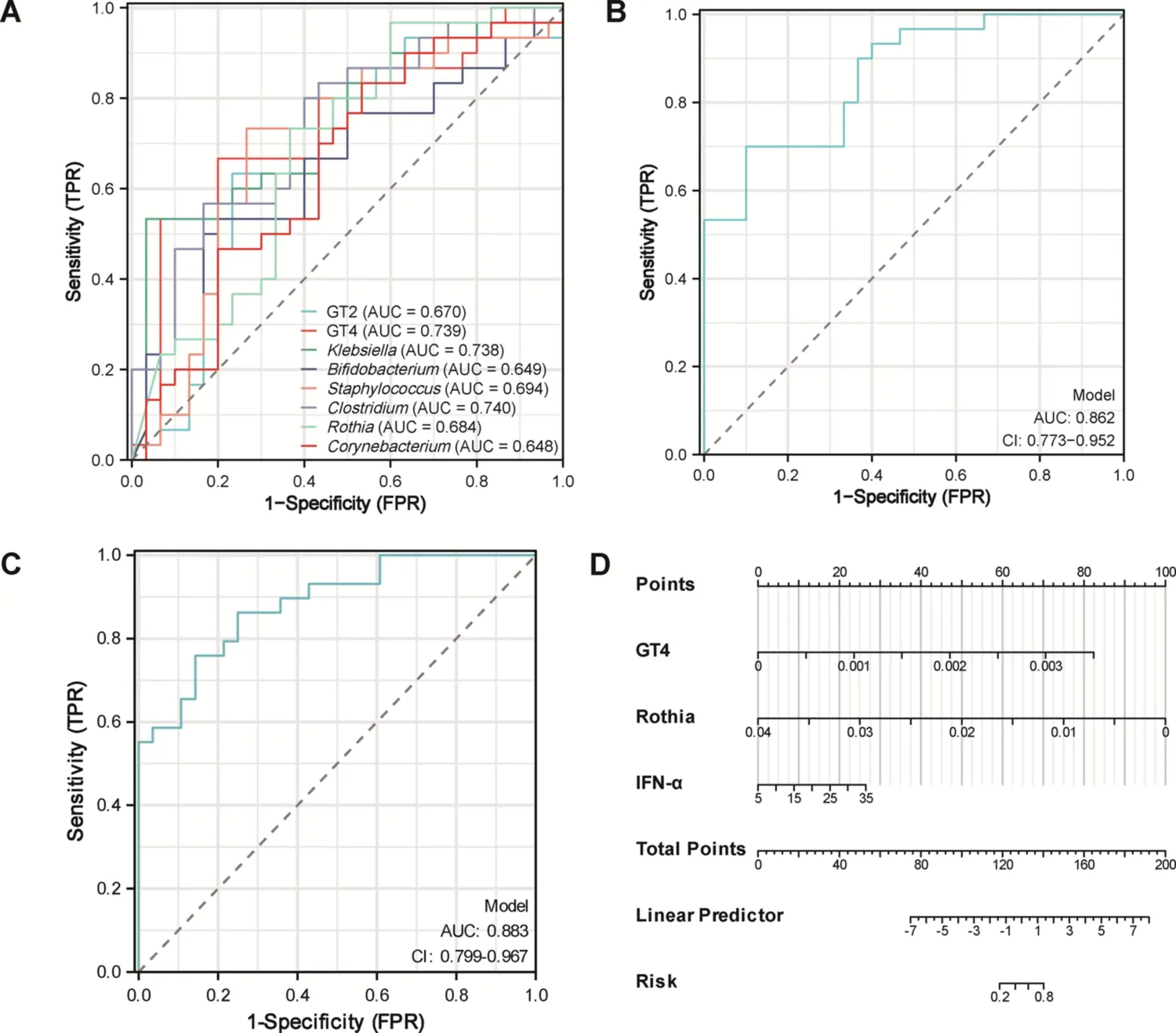
Construction and evaluation of microbiome- and immune-based predictive models for NLE risk. ROC curves for individual differentially abundant microbial genera, GT2, and GT4 in discriminating NLE (*n* = 30) from No-NLE (*n* = 30). (B) ROC curve for the combined microbial-functional model incorporating differential genera, GT2, and GT4. (C) ROC curve for the integrated model containing GT4, Klebsiella, Rothia, and IFN-*α*, selected by LASSO regression using the lambda.1se criterion. (D) Nomogram derived from multivariable logistic regression for estimating the probability of NLE. NLE, neonatal lupus erythematosus; ROC, receiver operating characteristic; No-NLE, anti-Ro/La antibody-exposed neonates without NLE; GT, glycosyltransferase; IFN-*α*, interferon-α; LASSO, least absolute shrinkage and selection operator; AUC, area under the receiver operating characteristic curve; CI, confidence interval.

Based on the differential microbial genera and functional features identified above, we further evaluated their predictive performance for NLE (Figure 5 **, Table S4**). ROC curve analyzes were performed for individual microbial genera and GT2 and GT4 features (Figure 5A). Individual microbial or functional markers showed moderate predictive performance, with AUC values ranging from 0.648 to 0.740. In contrast, the combined model integrating differential microbial genera with GT2 and GT4 achieved improved discrimination, with an AUC of 0.862 (95% CI: 0.773–0.952) (Figure 5B). To further identify integrated microbial and immune predictors, differential metagenomic features were combined with immune parameters and subjected to LASSO regression. The lambda.1se criterion identified GT4, *Klebsiella*, *Rothia*, and IFN-*α* as variables significantly associated with NLE occurrence (**Figure S3, Table S5**). A combined model incorporating these variables further improved predictive performance, achieving an AUC of 0.883 (95% CI: 0.799–0.967), with a sensitivity of 76.67% and specificity of 86.67% (Figure 5C). Multivariable logistic regression showed that GT4 and IFN-*α* were positively associated with NLE, whereas *Rothia* showed an inverse association (**Table S6**). After adjustment for gestational age, delivery mode, and feeding practices, GT4 (OR = 4.218, 95%CI = 1.097–16.212, *P* = 0.036), *Rothia* (OR = 0.173, 95%CI = 0.031–0.982, *P* = 0.046), and IFN-*α* (OR = 1.153, 95%CI = 1.003–1.325, *P* = 0.045) remained independently associated with NLE (Table 1). A nomogram based on these variables further demonstrated their potential utility for NLE risk prediction (Figure 5D).

**Table 1.** Logistic regression analysis of factors associated with NLE among anti-Ro/La antibody-exposed neonates.

Characteristics	Univariate analysis		Multivariate analysis	
	Odds ratio (95% CI)	*P* value	Odds ratio (95% CI)	*P* value
Gestational age	1.040 (0.845–1.281)	0.711	0.999 (0.722–1.381)	0.995
Cesarean section	1.152 (0.406–3.263)	0.791	1.288 (0.203–8.178)	0.788
Breastfeeding	0.874 (0.316–2.418)	0.795	1.036 (0.236–4.553)	0.963
GT4	2.442 (1.225–4.869)	0.011	4.218 (1.097–16.212)	0.036
*Klebsiella*	4.010 (1.587–10.130)	0.003	1.479 (0.502–4.360)	0.478
*Rothia*	0.201 (0.047–0.863)	0.031	0.173 (0.031–0.982)	0.046
Interferon-α	1.238 (1.096–1.398)	<0.001	1.153 (1.003–1.325)	0.045

Data are from 60 anti-Ro/La antibody-exposed neonates (30 No-NLE and 30 NLE). Data are presented as odds ratios with 95% confidence intervals (CIs). Multivariable logistic regression included the variables shown in the table. A two-sided *P* < 0.05 was considered statistically significant.

Abbreviations: NLE, neonatal lupus erythematosus; No-NLE, anti-Ro/La antibody-exposed neonates without NLE; CI, confidence interval; GT, glycosyltransferase.

### 3.7. Gut microbiome–derived factors amplify anti-Ro–dependent IFN-*α* responses

**Figure 6.**
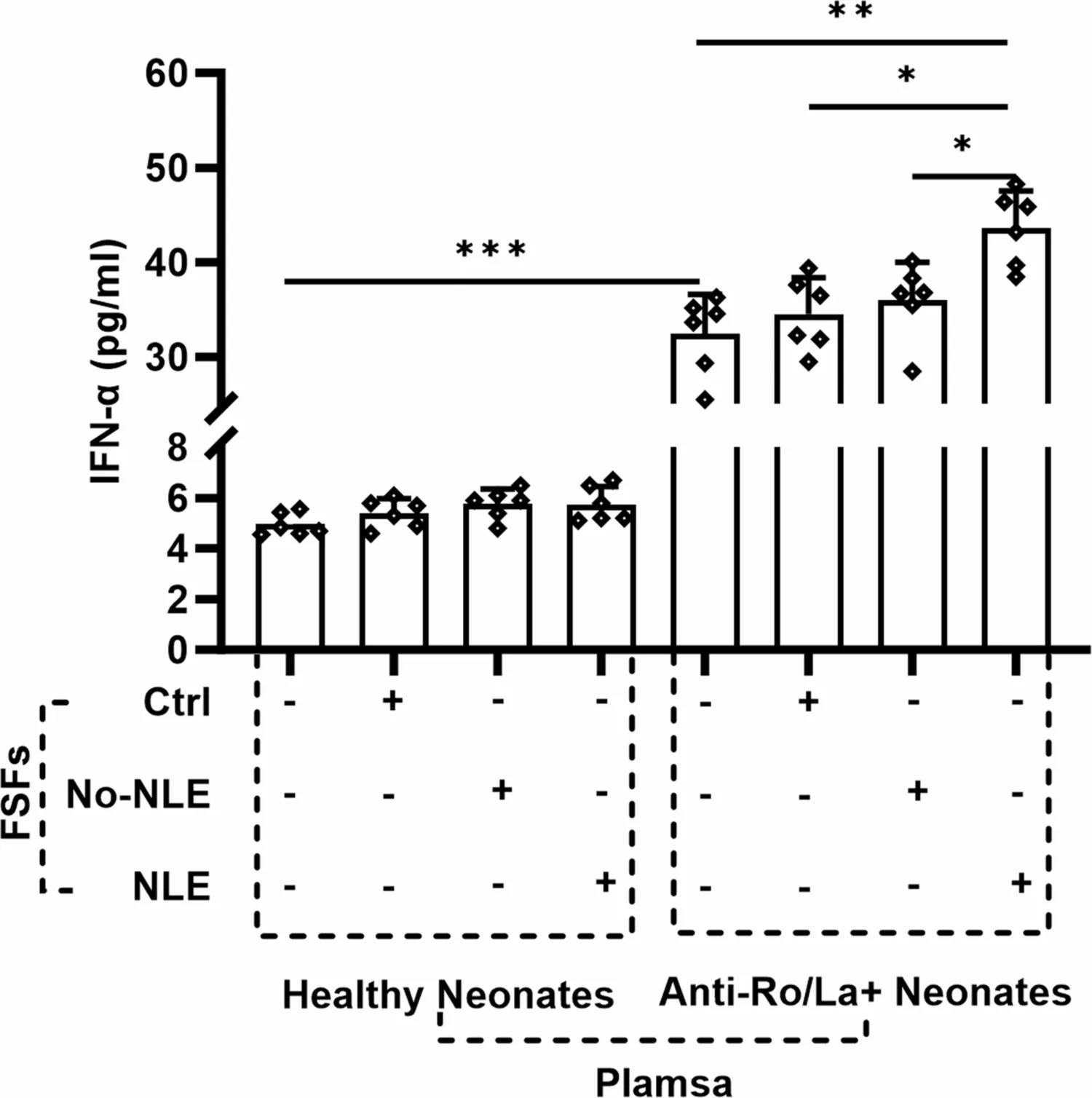
Effects of fecal soluble factors on anti-Ro/La-dependent IFN-*α* production by neonatal PBMCs in vitro. Peripheral blood mononuclear cells (PBMCs) from six independent healthy neonatal cord-blood donors were stimulated with phosphate-buffered saline (PBS) or pooled fecal filtrates from healthy controls, NLE, or No-NLE groups in the presence of pooled healthy neonatal or anti-Ro/La-positive plasma and necrotic cell supernatants. IFN-*α* levels were measured after 24 h. Statistical significance was assessed using paired two-tailed t-tests. IFN-*α*, interferon-α; NLE, neonatal lupus erythematosus; No-NLE, anti-Ro/La antibody-exposed neonates without NLE; *P < 0.05, **P < 0.01, ***P < 0.001.

To further examine whether gut microbiome–derived factors functionally modulate antibody-dependent immune responses, we performed in vitro assays to assess IFN-*α* production by PBMCs under different plasma and fecal filtrates. Previous studies have shown that anti-Ro antibodies can induce IFN-*α* production by PBMCs in vitro.^23^ Consistent with these findings, our in vitro functional assays (Figure 6) demonstrated that anti-Ro-positive plasma significantly induced IFN-*α* production in PBMCs derived from healthy neonates (*P* < 0.05). Under anti-Ro-positive plasma stimulation, fecal soluble factors derived from NLE neonates further enhanced IFN-*α* production compared with blank control, healthy control, and No-NLE groups (*P* < 0.05). In contrast, under healthy plasma conditions, IFN-*α* levels remained low, with no significant differences among fecal supernatant groups (*P* > 0.05).

### 3.8. Plasma metabolomic alterations and the effect of butyrate supplementation on anti-Ro/La-associated IFN-α activation

**Figure 7.**
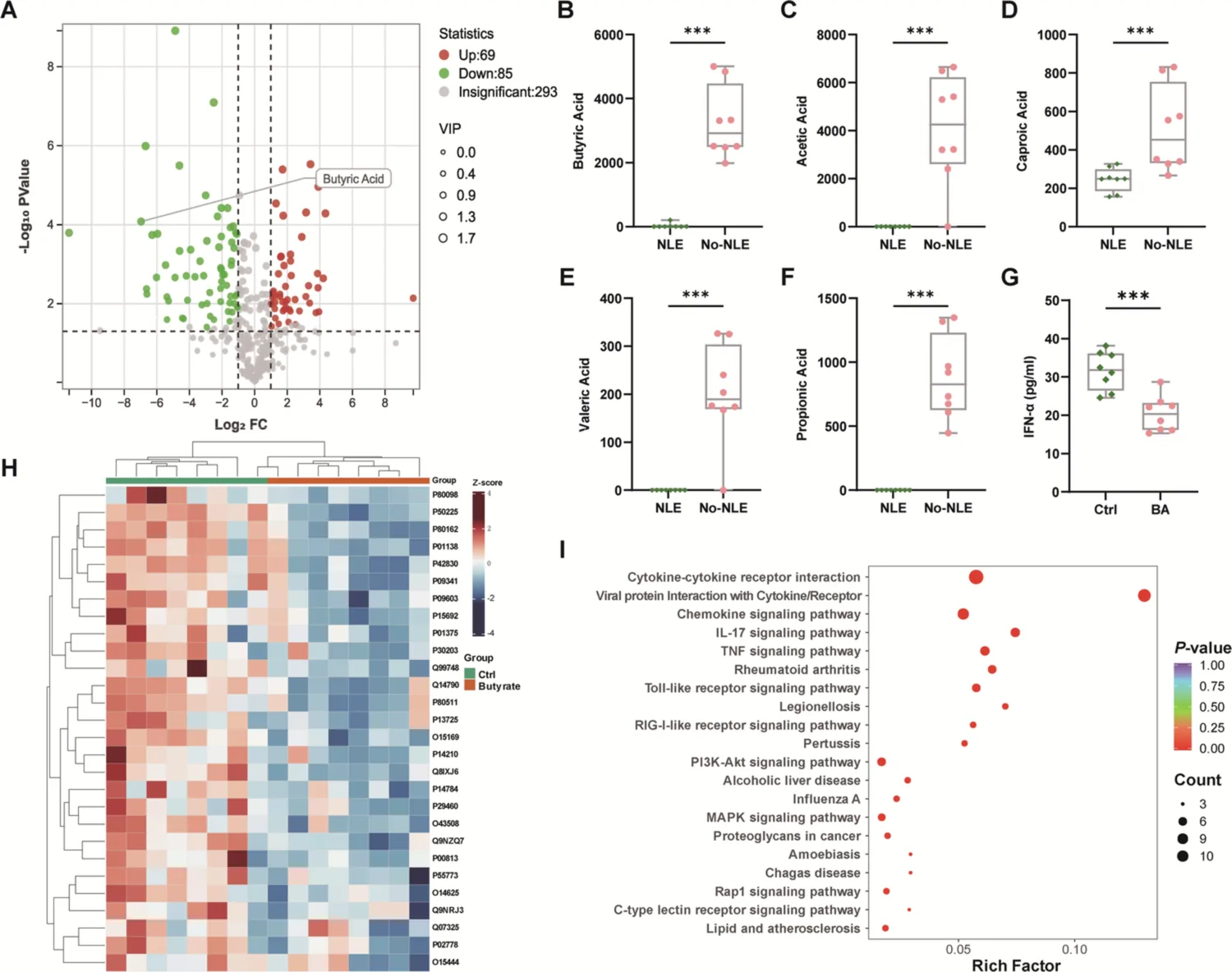
Plasma metabolomic alterations and butyrate-mediated suppression of anti-Ro/La-associated IFN-*α* responses. Volcano plot showing differential plasma metabolites between the NLE and No-NLE groups (*n* = 8 per group). Differential metabolites were defined by a variable importance in projection (VIP) score > 1 and a fold change ≥ 2 or ≤ 0.5. (B–F) Plasma concentrations of short-chain fatty acids (SCFAs), including butyric acid, acetic acid, caproic acid, valeric acid, and propionic acid. Two-group comparisons were performed using two-sided t-tests or Wilcoxon rank-sum tests according to data distribution. (G) IFN-*α* production by neonatal PBMCs stimulated with pooled anti-Ro/La-positive plasma and necrotic cell supernatant after treatment with vehicle or 1 mmol/L sodium butyrate for 24 h. PBMCs were obtained from eight independent healthy neonatal umbilical cord-blood donors. PBMCs from each donor were tested under both vehicle-control and sodium-butyrate conditions. Statistical significance was assessed using paired two-tailed t-tests. (H) Differentially expressed inflammation-related proteins quantified using the Olink Target 96 Inflammation panel after sodium butyrate treatment. (I) KEGG pathway enrichment analysis of proteins altered following sodium butyrate treatment. IFN-*α*, interferon-α; NLE, neonatal lupus erythematosus; No-NLE, anti-Ro/La antibody-exposed neonates without NLE; PBMC, peripheral blood mononuclear cell; KEGG, Kyoto Encyclopedia of Genes and Genomes; ***P < 0.001.

To determine whether gut microbiome functional alterations were accompanied by systemic metabolic changes, plasma metabolomic profiling was performed in a subset of NLE and No-NLE neonates. Plasma metabolomic profiles differed significantly between the two groups, with pathway alterations consistent with microbiome-associated functional changes (**Figure S4**). Notably, SCFA-related metabolites were broadly reduced in NLE neonates, including acetic acid, propionic acid, butyric acid, valeric acid, and caproic acid, with butyrate showing the most pronounced decrease (Figure 7A–F). To assess the functional relevance of reduced butyrate levels, butyrate supplementation was performed in the anti-Ro/La-positive PBMC stimulation model. Butyrate treatment significantly reduced IFN-*α* production compared with vehicle control (Figure 7G). Olink proteomic analysis identified 28 inflammation-related proteins downregulated after butyrate treatment, including C-X-C motif chemokine ligand 10 (CXCL10), adenosine deaminase (ADA), programmed death-ligand 1 (PD-L1), C-C motif chemokine ligand 23 (CCL23), and C-X-C motif chemokine ligand 6 (CXCL6) (Figure 7H). KEGG enrichment analysis showed that these proteins were mainly involved in cytokine–cytokine receptor interaction, chemokine signaling, interleukin 17 (IL-17) signaling, and tumor necrosis factor (TNF) signaling pathways (Figure 7I).

## 4. Discussion

Within the perinatal immune context of maternal anti-Ro/La antibody exposure, this study investigated how early-life gut microbial characteristics and microbiota-associated metabolic signals may influence the heterogeneous clinical expression of neonatal lupus erythematosus. By integrating shotgun metagenomics, plasma metabolomics, immune profiling, and functional validation assays, we identified coordinated alterations in microbial composition, microbial functional capacity, circulating metabolites, and immune responsiveness associated with NLE. Importantly, our findings support a model in which early-life microbial alterations may influence the threshold of antibody-dependent immune activation rather than acting as independent disease triggers. The identified microbiota–butyrate–type I interferon pathway provides a potential mechanistic framework explaining why only a subset of anti-Ro/La-exposed neonates develop clinical manifestations.

Early life represents a critical developmental window during which gut microbial colonization and immune maturation occur simultaneously; microbial communities provide essential signals for immune education, tolerance establishment, and inflammatory regulation.^24^ ^,^ ^25^ In our cohort, anti-Ro/La-exposed neonates exhibited altered microbial community structures shortly after birth, with progressive divergence from No-NLE to NLE groups. Given the comparable perinatal clinical characteristics among groups, these findings suggest that maternal autoantibody exposure may interact with early microbial development and contribute to distinct immune–microbiome states.^26-28^ The intermediate microbial profile observed in No-NLE neonates further indicates that antibody exposure alone may not be sufficient to induce clinical disease, whereas additional microbial and metabolic perturbations may contribute to disease manifestation.^29^ ^,^ ^30^

At the taxonomic level, NLE neonates demonstrated enrichment of *Klebsiella* accompanied by depletion of potentially immunoregulatory commensals, including *Bifidobacterium* and *Rothia*. These microbial shifts represent an ecological transition toward a community configuration associated with reduced immune regulatory potential. *Bifidobacterium* is a major early-life commensal involved in microbial maturation and immune homeostasis,^31^ ^,^ ^32^ whereas expansion of *Klebsiella* and related opportunistic taxa has been observed under inflammatory or immune-disrupted conditions.^33^ ^,^ ^34^ However, the contribution of individual microbial taxa to NLE development remains uncertain. Rather than representing direct pathogenic organisms, these taxa may reflect or contribute to a microbial ecosystem with altered immunological signaling capacity. The distinct but partially overlapping microbial profiles between No-NLE and NLE groups further suggest that clinical manifestation may depend on the interaction between antibody exposure, microbial ecology, and host immune susceptibility.

Beyond compositional changes, our data underscore microbiome functional reprogramming as a prominent feature associated with NLE. In particular, the convergence between metagenomic functional shifts and plasma metabolic signatures suggests that microbial remodeling in NLE is accompanied by coordinated systemic metabolic alterations, rather than remaining confined to the intestinal ecosystem. At the pathway level, the enrichment of carbohydrate utilization-related functions, together with pronounced changes in carbohydrate-active enzyme profiles, especially the glycosyltransferase families GT2 and GT4, highlights a plausible functional interface linking microbial glycan biosynthesis and surface remodeling to mucosal host–microbe interactions. As suggested in infant cohort studies, perturbations in microbial functional capacities may be more informative for immune susceptibility than taxonomic composition alone.^35^ Collectively, these findings support a model in which early-life dysbiosis in anti-Ro-exposed neonates is accompanied by functional remodeling, potentially shaping an immune-permissive biochemical context for downstream immune activation.

Type I interferon signaling is a central pathway implicated in NLE immunopathogenesis.^36^ Under homeostatic conditions, it contributes to immune tolerance, whereas dysregulated or excessive activation can shift this pathway toward immunopathology.^37^ ^,^ ^38^ In our cohort, NLE neonates exhibited increased circulating IFN-*α* and IgG levels together with reduced complement C4, indicating enhanced immune activation and impaired immune regulation.^39^ ^,^ ^40^ Importantly, functional assays provided evidence that microbiota-associated soluble factors may modulate the magnitude of antibody-dependent interferon responses. Fecal filtrates derived from NLE neonates did not directly induce strong IFN-*α* production but significantly enhanced IFN-*α* secretion by neonatal peripheral blood mononuclear cells in the presence of anti-Ro/La-positive plasma. These findings suggest that microbial-derived signals may function as immune amplifiers, lowering the threshold for autoantibody-mediated inflammatory activation. Consistent with this model, plasma metabolomic analysis demonstrated broad reductions in microbiota-associated SCFAs, with butyrate representing a potential regulatory mediator. Butyrate is recognized as an important microbial metabolite involved in immune regulation through effects on cellular metabolism, epigenetic regulation, and inflammatory signaling.^41^ ^,^ ^42^ In our experimental system, sodium butyrate supplementation suppressed anti-Ro/La-associated IFN-*α* production and reduced inflammatory protein responses involving cytokine, chemokine, IL-17, and TNF-related pathways. Together, these findings support a model in which reduced microbiota-associated butyrate availability may contribute to excessive type I interferon activation under conditions of maternal autoantibody exposure. While these findings are consistent with prior evidence linking SCFAs to the restraint of inflammatory programs and promotion of regulatory immune pathways, the precise mechanisms and their relevance to neonatal autoantibody exposure warrant further investigation.

From a translational perspective, the integrated microbial and immune signatures identified in this study may provide a framework for biological stratification of anti-Ro/La-exposed neonates. Although the combined microbial, functional, and immune features demonstrated potential discriminatory value between NLE and No-NLE groups, validation in larger independent cohorts is required before clinical application. Beyond biomarker development, our findings raise the possibility that microbiota-derived metabolites may represent potential intervention targets. Modulation of microbial communities or restoration of beneficial metabolites such as butyrate may theoretically influence immune activation thresholds in susceptible neonates. However, given the complexity of neonatal immune development and the absence of in vivo intervention studies, such therapeutic implications remain exploratory.

Several limitations should be considered. First, although this multicenter study included longitudinal clinical follow-up, the case–control design limits causal inference. The observed associations may reflect bidirectional interactions between immune activation and microbial remodeling.^43^ Second, NLE classification was based on established clinical manifestations together with maternal or neonatal anti-SSA/Ro and/or anti-SSB/La positivity. Subclinical, transient, or atypical manifestations may therefore have been under-recognized, and some inter-center variation in clinical ascertainment cannot be completely excluded despite standardized follow-up, blinded clinical assessment, and multidisciplinary adjudication. In addition, the GT4–Klebsiella–Rothia–IFN-*α* predictive signature was derived and evaluated within the same multicenter cohort and requires prospective external validation before being considered for clinical diagnostic or risk-stratification purposes. Third, despite comprehensive clinical characterization, residual confounding related to maternal immune status, genetic susceptibility, and environmental exposures cannot be completely excluded. Moreover, the available sample size was sufficient primarily to detect moderate-to-large effects, and smaller associations may have remained undetected. Fourth, although metagenomic functional profiles and plasma metabolomic alterations demonstrated concordant patterns, microbiota-derived metabolites were not directly quantified in fecal samples. Therefore, the relationship between microbial functional changes and systemic metabolite availability requires further validation. Finally, the in vitro butyrate supplementation experiments support a potential regulatory role of butyrate but cannot fully reproduce the complexity of intestinal microbial metabolism and neonatal immune environments. Future studies incorporating longitudinal multi-omics profiling, external validation cohorts, and mechanistic models will be necessary to establish causal relationships.^44^

In conclusion, this study identifies a microbiota–butyrate–type I interferon pathway associated with NLE development following maternal anti-Ro antibody exposure. We demonstrate that anti-Ro exposure is accompanied by early-life gut microbial dysbiosis, functional remodeling of microbial carbohydrate metabolism, altered systemic immune profiles, and reduced microbiota-associated metabolites. These alterations are associated with enhanced IFN-*α* responses and provide microbial and immunological signatures with potential value for NLE risk stratification. Although causal relationships require further validation, our findings highlight the importance of early-life microbiome–immune interactions in shaping neonatal immune responses under maternal autoantibody exposure and provide a foundation for future mechanistic and translational studies.

## Supplementary Material

Supplementary Material 2_Supplementary Data__Clean.docx

## Data Availability

The raw sequencing data generated during this study have been deposited in the National Center for Biotechnology Information (NCBI) Sequence Read Archive (SRA) under BioProject accession number PRJNA1414141 (https://www.ncbi.nlm.nih.gov/bioproject/PRJNA1414141). All other data generated during this study are included in this article and its Supplementary Material.

## Abbreviations

ABC: ATP-binding cassette
ACE: Abundance-based coverage estimator
ADA: Adenosine deaminase
AUC: Area under the receiver operating characteristic curve
C3: Complement component 3
C4: Complement component 4
CAZy: Carbohydrate-Active enZYmes database
CAZyme: Carbohydrate-active enzyme
CCL23: C-C motif chemokine ligand 23
CI: Confidence interval
Ctrl: Control group
CXCL6: C-X-C motif chemokine ligand 6
CXCL10: C-X-C motif chemokine ligand 10
DNBSEQ: DNA nanoball sequencing
DNA: Deoxyribonucleic acid
ELISA: Enzyme-linked immunosorbent assay
FASTQ: Sequence data format containing nucleotide sequences and quality scores
GH: Glycoside hydrolase
GH4: Glycoside hydrolase family 4
GT: Glycosyltransferase
GT2: Glycosyltransferase family 2
GT4: Glycosyltransferase family 4
IFN-α: Interferon-α
IgA: Immunoglobulin A
IgG: Immunoglobulin G
IgM: Immunoglobulin M
IL-17: Interleukin 17
KEGG: Kyoto Encyclopedia of Genes and Genomes
LASSO: Least absolute shrinkage and selection operator
LCA: Lowest common ancestor
LDA: Linear discriminant analysis
LEfSe: Linear discriminant analysis effect size
NCBI: National Center for Biotechnology Information
NMDS: Non-metric multidimensional scaling
NLE: Neonatal lupus erythematosus
No-NLE: Anti-Ro/La antibody-exposed neonates without NLE
NPX: Normalized protein expression
NR: Nonredundant
OPLS-DA: Orthogonal partial least-squares discriminant analysis
PBMC: Peripheral blood mononuclear cell
PBS: Phosphate-buffered saline
PCoA: Principal coordinates analysis
PD-L1: Programmed death-ligand 1
PE150: Paired-end 150-base-pair reads
PERMANOVA: Permutational multivariate analysis of variance
ROC: Receiver operating characteristic
rRNA: Ribosomal RNA
SCFA: Short-chain fatty acid
SRA: Sequence Read Archive
SSA: Sjögren syndrome-related antigen A
SSB: Sjögren syndrome-related antigen B
TNF: Tumor necrosis factor
UHPLC-HRMS: Ultra-high-performance liquid chromatography coupled with high-resolution mass spectrometry
VIP: variable importance in projection
