# Supplementary material for: Gut microbiota functional remodeling and butyrate depletion amplify anti-Ro/La antibody-driven type I interferon activation in neonatal lupus
Source: Gut Microbes. 2026 Sep 16;18(1):2728464. doi: 10.1080/19490976.2026.2728464 (PMC13596901; doi:10.1080/19490976.2026.2728464)
Supplement: Supplementary file 1 [file KGMI_A_2728464_SM7458.docx]

**SUPPLEMENTARY INFORMATION**

**Corresponding manuscript:** Gut microbiota functional remodeling and butyrate depletion amplify anti-Ro/La antibody-driven type I interferon activation in neonatal lupus

Authors: Wenqiang Sun ^1^, Yihui Li ^1^, Xue Liu ^1^, Shuyang Yu ^1^, Wenmei Li ^1^, Huawei Wang, Haifeng Geng, Lili Li, Jinhui Hu, Jie Huo, Wanyan Zhang, Jing Fu, Xinyun Jin, Heng Li, Xihui Zhou ^*^, Xueping Zhu ^*^

^1^ These authors contributed equally.

* Corresponding author

**APPENDIX A**

**Fecal microbial DNA extraction, metagenomic sequencing, and bioinformatic analysis**

***DNA extraction and metagenomic sequencing***

Genomic DNA was extracted from neonatal fecal samples. DNA concentration and integrity were assessed using a Qubit fluorometer and the Qsep400 system, respectively. Samples meeting the quality-control criteria were used for metagenomic library preparation. DNA was enzymatically fragmented to approximately 300 base pairs, followed by end repair, A-tailing, adaptor ligation, purification, and polymerase chain reaction amplification. After library quality assessment, 150-base-pair paired-end sequencing (PE150) was performed on the DNA nanoball sequencing (DNBSEQ) high-throughput sequencing platform.

***Sequence processing, gene catalog construction, and taxonomic annotation***

Raw reads were processed using fastp to remove adaptor sequences and low-quality reads. Reads were excluded when more than 40% of bases had quality scores below 15, when more than five ambiguous bases were present, or when the read length was shorter than 15 base pairs. Host-derived reads were removed by alignment to the human reference genome using Bowtie2. The resulting clean reads were assembled de novo using MEGAHIT, and contigs of at least 500 base pairs were retained. Assembly quality was evaluated using QUAST. Genes were predicted using MetaGeneMark, and a nonredundant gene catalog was generated using CD-HIT. Predicted genes shorter than 100 nucleotides were removed, and sequences were clustered at 95% identity and 90% alignment coverage. Gene abundance was quantified using Salmon and expressed as transcripts per million (TPM). Unigenes supported by no more than two mapped reads across all samples were excluded from downstream analyses. For taxonomic annotation, predicted protein sequences were aligned using DIAMOND against bacterial, fungal, archaeal, and viral entries in the National Center for Biotechnology Information nonredundant protein (NCBI NR) database (release 2022.05). Taxonomy was assigned using the lowest common ancestor algorithm, and relative abundance was calculated at the phylum, family, genus, and species levels.

***Microbial diversity and functional analyses***

Alpha diversity was evaluated using the abundance-based coverage estimator (ACE), Chao1, Shannon, and Simpson indices. Overall community structure was assessed using principal coordinates analysis (PCoA) and non-metric multidimensional scaling (NMDS) based on Bray-Curtis dissimilarities; between-group differences in beta diversity were tested using permutational multivariate analysis of variance (PERMANOVA). Differentially abundant taxa were identified using linear discriminant analysis effect size (LEfSe), with a linear discriminant analysis (LDA) score > 3.0 and *P* < 0.05. Genus-level microbial co-occurrence networks were constructed from relative-abundance profiles. For functional analysis, unigenes were annotated against the Kyoto Encyclopedia of Genes and Genomes (KEGG) database (version 5.0). Carbohydrate-active enzyme (CAZyme) families were annotated using the Carbohydrate-Active enZYmes (CAZy) database (release 2024.8). Overall CAZyme profiles were visualized using PCoA based on Bray–Curtis dissimilarities, and differential CAZyme families were identified by LEfSe using an LDA score > 2.0 and *P* < 0.05.

**SUPPLEMENTARY TABLES**

**Table S1. Baseline neonatal and maternal clinical characteristics**

| **Characteristics** | **Control** | | **No-NLE** | | **NLE** | ***P1*** | ***P2*** |
| --- | --- | --- | --- | --- | --- | --- | --- |
|  | (n=30) | | (n=30) | | (n=30) |  |  |
| Neonatal characteristics | | | | | | | |
| Male sex, n (%) | 16 (53.3) | 17 (56.7) | | 15 (50.0) | 1.000 | 0.605 | |
| Gestational age, weeks | 36.51 ± 1.43 | 36.11 ± 2.50 | | 36.21 ± 2.63 | 0.399 | 0.881 | |
| Birth weight, g | 2467.83 ± 252.19 | 2380.90 ± 556.81 | | 2329.33 ± 681.31 | 0.306 | 0.749 | |
| Cesarean delivery, n (%) | 15 (50.0) | 18 (60.0) | | 19 (63.3) | 0.291 | 0.791 | |
| Premature rupture of membranes, n (%) | 5 (16.7) | 4 (13.3) | | 4 (13.3) | 0.976 | 0.676 | |
| Postnatal asphyxia, n (%) | 1 (3.3) | 2 (6.7) | | 2 (6.7) | 0.661 | 1.000 | |
| Breastfeeding, n (%) | 16 (53.3) | 14 (46.7) | | 13 (43.3) | 0.456 | 0.795 | |
| Maternal characteristics | | | | | | | |
| Maternal age, years | 28.00 ± 3.50 | 28.26 ± 3.56 | | 29.00 ± 3.06 | 0.330 | 0.335 | |
| Maternal autoimmune disease | | | | | | | |
| SLE, n (%) | — | 15 (50.0) | | 14 (46.7) | — | 0.796 | |
| Sjögren syndrome, n (%) | — | 11 (36.7) | | 9 (30.0) | — | 0.584 | |
| Mixed connective tissue disease, n (%) | — | 0 (0.0) | | 1 (3.3) | — | 1.000 | |
| Isolated autoantibody positivity, n (%) | — | 4 (13.3) | | 8 (26.7) | — | 0.333 | |
| Coexisting SLE and Sjögren syndrome, n (%) | — | 0 (0.0) | | 2 (6.7) | — | 0.492 | |
| Antirheumatic medication during pregnancy | | | | | | | |
| Glucocorticoids, n (%) | — | 21 (70.0) | | 24 (80.0) | — | 0.371 | |
| Hydroxychloroquine, n (%) | — | 17 (56.7) | | 17 (56.7) | — | 1.000 | |
| Low-molecular-weight heparin, n (%) | — | 2 (6.7) | | 6 (20.0) | — | 0.255 | |
| Aspirin, n (%) | — | 3 (10.0) | | 4 (13.3) | — | 1.000 | |
| Immunosuppressants, n (%) | — | 3 (10.0) | | 4 (13.3) | — | 1.000 | |

Data are presented as mean ± standard deviation (SD) or n (%). *P1* compares all anti-Ro/La antibody-exposed neonates (No-NLE and NLE combined) with healthy controls; *P2* compares NLE with No-NLE. Continuous variables were analyzed using two-sided t tests, as appropriate, and categorical variables were analyzed using chi-square or Fisher’s exact tests. An em dash indicates that the comparison was not applicable or was not performed. NLE, neonatal lupus erythematosus; No-NLE, anti-Ro/La antibody-exposed neonates without NLE; SLE, systemic lupus erythematosus.

**Table S2. Maternal and neonatal anti-SSA/Ro and anti-SSB/La antibody reactivity grades by neonatal lupus status**

| **Antibody reactivity** | **No-NLE (n = 30)** | **NLE (n = 30)** | ***P* value (trend)** |
| --- | --- | --- | --- |
| **Maternal anti-SSA/Ro** |  |  | 0.027 |
| Negative | 2 (6.7) | 0 (0.0) |  |
| + | 13 (43.3) | 8 (26.7) |  |
| ++ | 11 (36.7) | 13 (43.3) |  |
| +++ | 4 (13.3) | 9 (30.0) |  |
| **Maternal anti-SSB/La** |  |  | 0.008 |
| Negative | 9 (30.0) | 3 (10.0) |  |
| + | 13 (43.3) | 11 (36.7) |  |
| ++ | 7 (23.3) | 10 (33.3) |  |
| +++ | 1 (3.3) | 6 (20.0) |  |
| **Neonatal anti-SSA/Ro** |  |  | 0.026 |
| Negative | 2 (6.7) | 0 (0.0) |  |
| + | 13 (43.3) | 9 (30.0) |  |
| ++ | 12 (40.0) | 12 (40.0) |  |
| +++ | 3 (10.0) | 9 (30.0) |  |
| **Neonatal anti-SSB/La** |  |  | 0.010 |
| Negative | 9 (30.0) | 3 (10.0) |  |
| + | 14 (46.7) | 12 (40.0) |  |
| ++ | 6 (20.0) | 10 (33.3) |  |
| +++ | 1 (3.3) | 5 (16.7) |  |

Antibody reactivity was reported by the participating clinical laboratories as negative, +, ++, or +++ and is shown as n (%). For trend analyses, categories were coded as negative = 0, + = 1, ++ = 2, and +++ = 3. Differences in the distribution of ordinal reactivity grades between the No-NLE and NLE groups were assessed using two-sided Cochran–Armitage trend tests. NLE, neonatal lupus erythematosus; No-NLE, anti-Ro/La antibody-exposed neonates without NLE.

**Table S3. Associations of anti-Ro/La antibody reactivity with neonatal microbial, functional, and immune features**

| **Antibody variable** | **Neonatal parameter** | **Spearman’s ρ** | ***P* value** |
| --- | --- | --- | --- |
| Anti-SSA/Ro grade | *Klebsiella* | 0.477 | <0.001 |
|  | *Bifidobacterium* | −0.441 | <0.001 |
|  | *Clostridium* | −0.325 | 0.011 |
|  | *Rothia* | −0.261 | 0.044 |
|  | *Corynebacterium* | 0.074 | 0.576 |
|  | Glycosyltransferase family 2 (GT2) | −0.414 | 0.001 |
|  | Glycosyltransferase family 4 (GT4) | 0.386 | 0.003 |
|  | interferon-α (IFN-α) | 0.522 | <0.001 |
| Anti-SSB/La grade | *Klebsiella* | 0.467 | <0.001 |
|  | *Bifidobacterium* | −0.344 | 0.007 |
|  | *Clostridium* | −0.302 | 0.019 |
|  | *Rothia* | −0.213 | 0.103 |
|  | *Corynebacterium* | 0.136 | 0.302 |
|  | GT2 | −0.239 | 0.069 |
|  | GT4 | 0.343 | 0.008 |
|  | IFN-α | 0.603 | <0.001 |
| Combined anti-Ro/La burden score | Klebsiella | 0.516 | <0.001 |
|  | Bifidobacterium | −0.413 | 0.001 |
|  | Clostridium | −0.333 | 0.009 |
|  | Rothia | −0.257 | 0.048 |
|  | Corynebacterium | 0.115 | 0.380 |
|  | GT2 | −0.344 | 0.008 |
|  | GT4 | 0.385 | 0.003 |
|  | IFN-α | 0.616 | <0.001 |

Analyses included all 60 anti-Ro/La antibody-exposed neonates (30 No-NLE and 30 NLE). Maternal anti-SSA/Ro and anti-SSB/La grades were coded as negative = 0, + = 1, ++ = 2, and +++ = 3. The combined maternal anti-Ro/La burden score was calculated as the sum of the anti-SSA/Ro and anti-SSB/La grade scores. Microbial genera are represented by relative abundances, GT2 and GT4 by metagenomic functional abundances, and IFN-α by serum concentrations. Correlations were assessed using Spearman’s rank correlation, and values are presented as Spearman’s rank-correlation coefficients (ρ) with unadjusted two-sided *P* values. NLE, neonatal lupus erythematosus; No-NLE, anti-Ro/La antibody-exposed neonates without NLE.

**Table S4. Discriminative performance of individual and combined microbial-functional markers for neonatal lupus erythematosus**

| **Marker or model** | **AUC** | **95% CI** | **Sensitivity, %** | **Specificity, %** |
| --- | --- | --- | --- | --- |
| Glycosyltransferase family 2 (GT2) | 0.670 | 0.527–0.813 | 66.67 | 80.00 |
| Glycosyltransferase family 4 (GT4) | 0.739 | 0.609–0.869 | 53.33 | 96.67 |
| *Klebsiella* | 0.738 | 0.608–0.867 | 50.00 | 83.33 |
| *Bifidobacterium* | 0.649 | 0.507–0.791 | 53.33 | 80.00 |
| *Staphylococcus* | 0.694 | 0.553–0.836 | 50.00 | 83.33 |
| *Clostridium* | 0.740 | 0.614–0.866 | 80.00 | 60.00 |
| *Rothia* | 0.684 | 0.547–0.822 | 73.33 | 63.33 |
| *Corynebacterium* | 0.648 | 0.506–0.790 | 83.33 | 46.67 |
| Combined microbial-functional model | 0.862 | 0.773–0.952 | 70.00 | 90.00 |

Receiver operating characteristic analyses were performed in 60 anti-Ro/La antibody-exposed neonates (30 No-NLE and 30 NLE). The combined microbial-functional model incorporated GT2, GT4, *Klebsiella*, *Bifidobacterium*, *Staphylococcus*, *Clostridium*, *Rothia*, and *Corynebacterium*. AUC, area under the receiver operating characteristic curve; CI, confidence interval; NLE, neonatal lupus erythematosus; No-NLE, anti-Ro/La antibody-exposed neonates without NLE.

**Table S5. Coefficients of candidate predictors from least absolute shrinkage and selection operator regression**

| **Variable** | **Coefficient at λ1se** | **Coefficient at λmin** |
| --- | --- | --- |
| Intercept | 0.20 | 0.38 |
| Glycosyltransferase family 2 (GT2) | 0 | 0 |
| Glycosyltransferase family 4 (GT4) | 0.17 | 0.57 |
| *Klebsiella* | 0.81 | 1.16 |
| *Bifidobacterium* | 0 | 0 |
| *Staphylococcus* | 0 | 0 |
| *Clostridium* | 0 | 0 |
| *Rothia* | −0.19 | −0.62 |
| *Corynebacterium* | 0 | 0 |
| Interferon-α (IFN-α) | 0.18 | 0.31 |
| Immunoglobulin G (IgG) | 0 | 0.02 |
| Complement component 4 (C4) | 0 | 0 |

Least absolute shrinkage and selection operator (LASSO) regression was applied to integrated microbial, metagenomic-functional, and immune variables in the 60 anti-Ro/La antibody-exposed neonates. λmin denotes the penalty parameter that minimized the mean cross-validated binomial deviance, whereas λ1se denotes the largest penalty parameter within 1 standard error of the minimum and yields a more parsimonious model. At λ1se, GT4, *Klebsiella*, *Rothia*, and IFN-α retained nonzero coefficients and were carried forward to multivariable logistic regression. NLE, neonatal lupus erythematosus.

**Table S6. Univariable and multivariable logistic regression of LASSO-selected factors associated with neonatal lupus erythematosus**

| **Predictor** | **Univariable analysis** | | **Multivariable analysis** | |
| --- | --- | --- | --- | --- |
|  | Odds Ratio (95% CI) | *P* value | Odds Ratio (95% CI) | *P* value |
| Glycosyltransferase family 4 (GT4) | 2.442 (1.225 – 4.869) | 0.011 | 4.054 (1.141 – 14.404) | 0.030 |
| *Klebsiella* | 4.010 (1.587 – 10.130) | 0.003 | 1.799 (0.640 – 5.056) | 0.265 |
| *Rothia* | 0.201 (0.047 – 0.863) | 0.031 | 0.171 (0.030 – 0.986) | 0.048 |
| Interferon-α (IFN-α) | 1.238 (1.096–1.398) | < 0.001 | 1.157 (1.013 – 1.322) | 0.031 |

Analyses included 60 anti-Ro/La antibody-exposed neonates (30 No-NLE and 30 NLE). The multivariable model simultaneously included the four predictors retained by LASSO at λ1se. The model shown here did not additionally adjust for perinatal covariates; the covariate-adjusted model including gestational age, delivery mode, and feeding practice is presented in Table 1 of the main manuscript. LASSO, least absolute shrinkage and selection operator; OR, odds ratio; CI, confidence interval; NLE, neonatal lupus erythematosus; No-NLE, anti-Ro/La antibody-exposed neonates without NLE.

**SUPPLEMENTARY FIGURES**

**Figure S1. Study design, sampling timeline, final group adjudication, and downstream analyses.** Pregnancies at potential risk of neonatal lupus erythematosus (NLE) were identified during the perinatal period, and eligible neonates were enrolled at birth. Peripheral blood and fecal samples were collected on postnatal days 7-10 before outcome adjudication; neonatal anti-SSA/Ro and anti-SSB/La reactivity was measured in serum collected during this period. All neonates were followed from birth to 6 months for NLE-related manifestations, after which final clinical adjudication and group classification were completed by clinicians blinded to the multi-omics results. For each confirmed NLE case, one anti-Ro/La-exposed neonate without NLE was selected at a 1:1 ratio according to gestational age and birth weight, while healthy controls were enrolled separately. Downstream metagenomic, metabolomic, immunological, and functional analyses were performed after final group assignment. Shotgun metagenomics included all three clinical groups (n = 30 per group), whereas plasma metabolomics included randomly selected NLE and No-NLE subcohorts (n = 8 per group). Fecal filtrate–PBMC assays used PBMCs from six independent healthy neonatal cord-blood donors, and butyrate intervention with Olink proteomics used PBMCs from eight donors under paired vehicle and sodium-butyrate conditions.

**Figure S2. Differential gut microbial genera across healthy controls, No-NLE neonates, and NLE neonates.**

(A) Linear discriminant analysis effect size (LEfSe) cladogram showing taxa that differed among healthy controls (Ctrl; n = 30), anti-Ro/La antibody-exposed neonates without neonatal lupus erythematosus (No-NLE; n = 30), and neonates with neonatal lupus erythematosus (NLE; n = 30). **(B)** Genus-level linear discriminant analysis (LDA) effect sizes for discriminatory taxa identified by LEfSe (LDA score > 3.0 and *P* < 0.05). **(C-K)** Relative abundances of *Clostridium* (C), *Collinsella* (D), *Corynebacterium* (E), *Clostridioides* (F), *Klebsiella* (G), *Staphylococcus* (H), *Streptococcus* (I), *Bifidobacterium* (J), and *Rothia* (K). Relative-abundance values were log_10_-transformed and shifted to positive values for visualization. Three-group comparisons were performed using the Kruskal-Wallis test followed by pairwise Wilcoxon rank-sum tests. ns, not significant; **P* < 0.05, ***P* < 0.01, and ****P* < 0.001.

**Figure S3. Least absolute shrinkage and selection operator selection of integrated microbial, functional, and immune predictors of neonatal lupus erythematosus.**

**(A)** Cross-validated binomial deviance plotted against the logarithm of the penalty parameter (λ) for the integrated candidate-predictor set. Points represent the mean cross-validated deviance and vertical bars represent ±1 standard error. The dashed vertical lines indicate λmin, which minimized mean deviance, and λ1se, the largest λ within 1 standard error of the minimum. Numbers above the plot indicate the number of nonzero coefficients. **(B)** Coefficient paths for candidate predictors across values of log(λ). Candidate variables comprised differential microbial genera, glycosyltransferase families, and serum immune parameters from 60 anti-Ro/La antibody-exposed neonates (30 No-NLE and 30 NLE). At λ1se, glycosyltransferase family 4 (GT4), *Klebsiella*, *Rothia*, and interferon-α (IFN-α) retained nonzero coefficients. LASSO, least absolute shrinkage and selection operator; NLE, neonatal lupus erythematosus; No-NLE, anti-Ro/La antibody-exposed neonates without NLE.

**Figure S4. Plasma metabolomic profiles of NLE and No-NLE neonates.**

(A) Orthogonal partial least squares discriminant analysis (OPLS-DA) score plot of global plasma metabolomic profiles in NLE and No-NLE neonates (n = 8 per group). **(B)** Volcano plot of metabolites detected in NLE relative to No-NLE. Each point represents one metabolite; red and green points indicate metabolites increased and decreased in NLE, respectively, and gray points indicate metabolites not meeting the differential criteria. In total, 69 metabolites were increased and 85 were decreased. Differential metabolites were defined by a variable importance in projection (VIP) score > 1 and a fold change ≥ 2 or ≤ 0.5. **(C)** Kyoto Encyclopedia of Genes and Genomes (KEGG) pathway-enrichment analysis of differential metabolites. **(D)** Heatmap of normalized, metabolite-wise scaled intensities for differential metabolites. Rows represent individual neonates and columns represent metabolites. NLE, neonatal lupus erythematosus; No-NLE, anti-Ro/La antibody-exposed neonates without NLE.
